# 1228. Outcomes Associated with Empiric Cefepime or Meropenem for Bloodstream Infections Caused by Ceftriaxone-Resistant, Cefepime-Susceptible *Escherichia coli* and *Klebsiella pneumoniae*

**DOI:** 10.1093/ofid/ofab466.1420

**Published:** 2021-12-04

**Authors:** Brian E Frescas, Christopher McCoy, James Kirby, Robert Bowden, Nicholas J Mercuro

**Affiliations:** Beth Israel Deaconess Medical Center, Boston, Massachusetts

## Abstract

**Background:**

Cefepime is a 4^th^ generation cephalosporin frequently used for empiric sepsis therapy. Dose- and MIC-dependent efficacy of cefepime is supported by the Clinical & Laboratory Standards Institute, however its use in infections due to extended-spectrum beta-lactamase-producing *Enterobacterales* is controversial. This study aims to compare outcomes in patients given empiric meropenem or cefepime for bloodstream infections (BSI) caused by ceftriaxone-resistant *E. coli* and *K. pneumoniae*.

**Methods:**

This single-center retrospective cohort included adults hospitalized from 2010 - 2020 and received empiric cefepime or meropenem for BSI caused by ceftriaxone-resistant *E. coli* or *K. pneumoniae*. In the cefepime group, only organisms with MIC ≤ 2 mg/L were included. Patients who received the empiric agent for < 48 hours, or received an additional active agent within 48 hours were excluded. The primary outcome was 30-day mortality; secondary outcomes were recurrent infection, readmission, and time to clinical stability. Chi-squared or Fisher’s exact was used for categorical variables and Mann-Whitney-U for continuous variables. Inverse probability treatment weighing was used to determine the impact of empirical therapy on clinical stability at 48 hours.

**Results:**

Fifty-four patients were included: 36 received empiric meropenem, 18 received cefepime. There were no significant differences in baseline severity of illness or comorbid conditions. Urinary source was less common in the meropenem group compared to cefepime (52.8 vs 83.8%, p=0.028) (Table 1). There was no difference in 30-day mortality between meropenem and cefepime (2.8 vs 11.1%, p = 0.255). More patients achieved clinical stability at 48 hours on empiric meropenem compared to cefepime (75 vs 44.4%, p = 0.027), and time to clinical stability was significantly shorter (median 21.3 vs 38.5 hours, p = 0.016). Most patients in the meropenem and cefepime groups completed definitive treatment with a carbapenem (88.9 vs 72.2%, p=0.142).

Table 1: Results

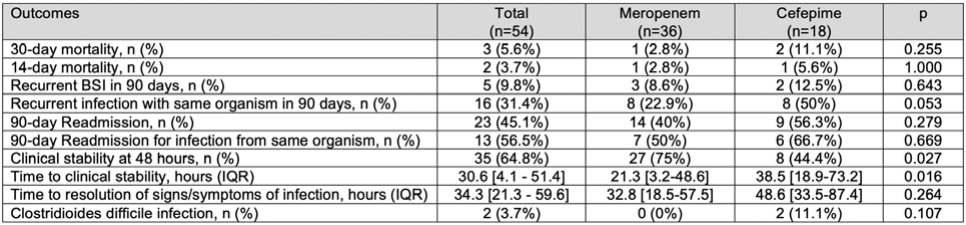

Summary of primary and secondary outcomes

**Conclusion:**

There was no difference in mortality between patients receiving empiric cefepime for BSI due to ceftriaxone-resistant *Enterobacterales,* with cefepime MIC ≤ 2 mg/L, compared to meropenem; however, time to clinical stability was significantly delayed.

**Disclosures:**

**James Kirby, MD, D(ABMM**), **First Light Biosciences** (Board Member)**TECAN, Inc.** (Research Grant or Support)

